# The Professional Classes Relief Fund

**Published:** 1915-06-05

**Authors:** 


### THE PROFESSIONAL CLASSES RELIEF FUND.

### Dr. Scharlieb on its Maternity Work.

At the invitation of Lady Northcote, a meeting was held at her house, 25 St. James's Place, S.W., on Tuesday, to consider the problem of the existing distress among members of various professional classes in consequence of the war. The chair was occupied by Major Leonard Darwin, President of the Professional Classes War Relief Council and of the Eugenics Education Society; and he was supported by the Right Hon. Sir John Simon, Sir John McClure, Sir A. Dyke Acland, and Dr. Mary Scharlieb.

The Chairman gave a brief sketch of the work which the Council had done from the commencement of the war in carrying out, according to the funds available, the needed help in alleviation of the distress experienced by large numbers of members of the professional classes. While it was felt that nothing should be allowed to stand in the way of succour in the fullest possible way for those who were wounded in battle, yet it was evident that real suffering and privation would be endured by those in professional positions. They usually suffered in silence. The Council had co-ordinated various philanthropic agencies, so as to ensure a proper co-operative activity. It was felt that such a Council was necessary in addition to a Government Sub-Committee, because the aims of the two were not quite identical, and a greater elasticity in working was permissible for such a Council than for a Government body. The aim of the Council of which he was Chairman was to prevent professional people from falling, in consequence of the war, to complete destitution, so that when the struggle was over there would not have been lost that power of rehabilitation which all desired. He paid a special tribute to the schoolmasters who were, without fee, continuing the education of children whose parents were badly hit by the war.

### The Council's Experience.

Sir John McClure spoke specially of the work done by the Economic and Legislation Committee and by the Finance Committee. The experience of the Council had been that it was the small professional people who had suffered most severely. The education of the young people at the present time was not the least of the many problems now facing us. To give small doles to many was thought not to be so effective as to help a comparatively few somewhat more. As an instance, a distinguished lady student of medicine had been enabled to complete her curriculum by means of a generous response to an advertisement which the Council inserted in newspapers. And while boys and girls were helped to join the ranks of wage-earners, others were discouraged from entering or continuing in certain professions in which mediocrity was most undesirable. In reference to the free continuation of education to girls and boys already at school, the Council had also made grants of pocket-money, so that such pupils should not feel the disparity between themselves and their associates. As the Council was now approaching the end of its funds, he made an eloquent appeal for renewed support.

Sir John Simon said the Council was rightly to be regarded as doing one of the pieces of work which a great self-conscious community in the throes of an unprecedented struggle should support. Though the war involved terrible economic and financial sacrifices for all classes, it acted very unequally upon the immediate prosperity of different individuals. At this time the country could not spare the energies of one man who worked with his hands, and there was need for his work to be organised. Neither could we spare a single sovereign belonging to those possessing wealth and having credit. But on the professional classes the benefits of extra remuneration were now least likely to fall. The work undertaken by this Council was work of national importance, and had calls for support from all.

### The Maternity Home, Prince's Gate.

Dr. Mary Scharlieb said she did not doubt that among all the other important branches of this war relief for the professional classes, nothing was more important than the maternity branch. It aimed at giving a home, medical attention, and skilled nursing to the wives of men whose careers had been damaged or broken, or at any rate whose professional income had temporarily suffered, owing to the war. In many instances the women who had applied to that department were the wives of young officers who had gone abroad, when an income of some hundreds a year was replaced by only the soldier's pay. The Council had been enabled to open this Maternity Home by the co-operation of many people. For instance, the executors of the estate of the late Mr. Pierpont Morgan had granted the use of two beautiful houses, Nos. 13 and 14 Prince's Gate, with delightful uninterrupted views both front and back, free of rent and rates, for the duration of the war—an enormous asset. And the medical men and women engaged there were giving their services freely, and the same was true of the nurses: the only salaried person was the matron. The patients were, for the period of their stay, given all the comforts they had been accustomed to. But more support was needed. The Chairman had pointed out that in many instances money could be provided by professional Associations, and one of the patients had been greatly helped by Lady Hope's Fund for Officers' Wives. There were many cases which they ought to help, but there was no institution or fund ready to contribute, and therefore the public had to be appealed to. It was a duty to contribute to this fund partly as an act of penitence and reparation, because in Germany especially, and to some extent in this kingdom, the war owed its existence to the exaltation of materialism.

The Chairman, in reference to Dr. Scharlieb's speech, said if it had only been on account of the twenty-six babies born in that Home, he would be proud to be Chairman of the institution. He wished to pay a tribute to the voluntary and salaried staff for the splendid work they had done. Only a visit would bring home to anyone the work which was being accomplished, as Lady Northcote could testify. This organisation was now spending £430 per week, and at first receipts came in faster than they were expended, so that at one time there was a sum of £3,000 in hand. But he regretted to say that if supplies did not come in faster than at present the work must come to an end in three months' time. The requirement for the coming year was a minimum of £25,000.

After a vote of thanks to Lady Northcote had been carried, the meeting terminated with an intimation that a loan exhibition of Whistler's works was in progress for the support of the fund at 144 to 146 Bond Street.

